# Molecular typing of *Streptococcus suis* strains isolated from diseased and healthy pigs between 1996-2016

**DOI:** 10.1371/journal.pone.0210801

**Published:** 2019-01-17

**Authors:** T. Louise Prüfer, Judith Rohde, Jutta Verspohl, Manfred Rohde, Astrid de Greeff, Jörg Willenborg, Peter Valentin-Weigand

**Affiliations:** 1 Institute for Microbiology, University of Veterinary Medicine, Hannover, Germany; 2 Central Facility for Microscopy, Helmholtz Centre for Infection Research, Braunschweig, Germany; 3 Wageningen Bioveterinary Research, Lelystadt, The Netherlands; Defense Threat Reduction Agency, UNITED STATES

## Abstract

*Streptococcus suis* is an economically important pathogen of pigs as well as a zoonotic cause of human disease. Serotyping is used for further characterization of isolates; some serotypes seem to be more virulent and more widely spread than others. This study characterizes a collection of German field isolates of *Streptococcus suis* from pigs dating from 1996 to 2016 with respect to capsular genes (*cps*) specific for individual serotypes and pathotype by multiplex PCR and relates results to the clinical background of these isolates. The most prominent finding was the reduction in prevalence of serotype-2/serotype-1/2 among invasive isolates during this sampling period, which might be attributed to widely implemented autogenous vaccination programs in swine against serotype 2 in Germany. In diseased pigs (systemically ill; respiratory disease) isolates of serotype-1/serotype-14, serotype-2/serotype-1/2, serotype 3 to 5 and 7 to 9 were most frequent while in carrier isolates a greater variety of *cps* types was found. Serotype-1/serotype-14 seemed to be preferentially located in joints, serotype 4 and serotype 3 in the central nervous system, respectively. The virulence associated extracellular protein factor was almost exclusively associated with invasive serotype-1/serotype-14 and serotype-2/serotype-1/2 isolates. In contrast, lung isolates of serotype-2/serotype-1/2 mainly harbored the gene for muramidase-released protein. Serotype 4 and serotype 9 isolates from clinically diseased pigs most frequently carried the muramidase-released protein gene and the suilysin gene. When examined by transmission electron microscopy all but one of the isolates which were non-typable by molecular and serological methods showed various amounts of capsular material indicating potentially new serotypes among these isolates. Given the variety of *cps* types/serotypes detected in pigs, not only veterinarians but also medical doctors should consider other serotypes than just serotype 2 when investigating potential human cases of *Streptococcus suis* infection.

## Introduction

*Streptococcus* (*S*.) *suis* is a facultative pathogen affecting humans, feral and domestic pigs. In Asia, where humans and pigs often live in close proximity, zoonotic epidemics with large numbers of human cases and even fatalities have been recorded in 1998 and 2005 [1]. *S*. *suis* is the most common cause of adult meningitis in Vietnam [2]. By contrast, in Western countries infections in humans usually occur only sporadically in those persons professionally occupied with keeping, handling or slaughtering pigs or processing their meat [1].

In pigs, asymptomatic colonization of the upper respiratory tract with *S*. *suis*, but also of the intestinal and genital tract, is common. On the other hand, *S*. *suis* is one of the economically most important pathogens in the pig industry causing primarily meningitis, arthritis and septicemia mainly in piglets and weaners [3, 4].

For first-line epidemiological discrimination of *S*. *suis* isolates, serotyping is useful. However, serotyping by co-agglutination is dependent on the availability and quality of sera, interpretation of results is somewhat subjective, and auto-, cross-, poly- and non-agglutination interfere with the analysis. These disadvantages may be avoided by a recently published two-step PCR-based serotyping method [5].

In humans, most clinical cases are reported to be caused by serotype 2 and serotype 14 isolates. However, in Europe there is a proportion of 37% of all published cases in which the serotype is unknown, worldwide this proportion is 23% [6]. Notably, some clinical laboratories focus specifically on detection of serotype 2 only.

For pigs, prevalence of different serotypes in diseased as well as in healthy animals is not well known for most countries. However, given the economic importance for the pig industry, the zoonotic potential of the pathogen and the fact that there is a substantial number of human cases that is undetermined by serotype [6, 7], it is of great interest for veterinary medicine as well as for human medicine to get more insights into serotype distribution of *S*. *suis* in pigs.

Therefore, in this study we applied the two-step multiplex PCR published by Okura et al. [5], which allows *cps* typing of all 35 serotypes (including the types no longer considered to be actually *S*. *suis*), for analysis of a collection of more than 500 German porcine *S*. *suis* isolates obtained from healthy and diseased pigs in 2015 and 2016. Furthermore, an older collection of 189 isolates from 1996–2004 was re-typed using the two-step multiplex PCR to evaluate possible changes in serotype distribution over time. Additionally, all 22 isolates from 2015–2016, that could not be *cps* typed by PCR or serotyped by agglutination, were further examined for expression of a capsule by transmission electron microscopy.

## Materials and methods

### Bacterial isolates

Reference strains for serotypes 1–34 were obtained from Christoph Baums, (University of Veterinary Medicine Hannover, Germany), Marcello Gottschalk (University of Montreal, Canada), and Hilde Smith (Wageningen University and Research Centre, The Netherlands). Field isolates were collected at the Institute for Microbiology at the University of Veterinary Medicine, Hannover, Germany. One collection comprised 522 strains isolated in 2015 and 2016 (collection B) and was compared to a collection of 189 isolates from 1996–2004 (collection A). Isolates were collected from diagnostic samples from live animals or after necropsy; no animals were euthanised specifically for this study. The sampling was part of the normal veterinary diagnostic investigation on a farm and as such was not for scientific purposes. All sampling was undertaken strictly according to the German animal welfare act. Isolate collections were curated and only one isolate of the same sero-/pathotype per farm was included in the analysis. The regional provenance of the isolates is inscribed into a map of Germany in the supporting information (S1 Fig). According to anamnestic information, all isolates were allocated into three groups: (1) invasive isolates from the central nervous system (CNS; cerebrospinal fluid or brain tissue) from pigs with typical clinical symptoms or from joints with arthritis or from heart, liver, spleen or blood; (2) respiratory disease isolates from pigs without signs of systemic spreading isolated from broncho-alveolar lavage fluid or lung tissue; (3) carrier isolates mainly from the upper respiratory tract (nose, tonsils) of healthy animals from farms without diagnosed streptococcal disease during the last two years. In collection A 70 isolates were from systemically ill pigs, 74 isolates from animals with respiratory diseases and 45 isolates from carrier animals; collection B comprised 353 invasive isolates, 117 respiratory and 52 carrier isolates. All isolates were stored at -80°C, thawed and passaged on Columbia sheep blood agar prior to analysis.

### Serotyping

Serotyping was done using the two-step multiplex PCR described by Okura et al. in 2014 [5]. This PCR identifies the serotype based on the serotype specific sequence differences in the *wzy*-gene of the *cps* gene cluster. In a first PCR, isolates are assigned to one of seven groups comprising several serotypes and in a second PCR the serotype within this group is determined. However, this method does not allow to distinguish serotype 2 and serotype 1/2 (designated hereinafter serotype-2/serotype-1/2) nor serotype 1 and serotype 14 (designated hereinafter serotype-1/serotype-14). Universal primers for the amplification of 16S rRNA genes were used as internal controls for all reactions. Finally, we included an in-house designed primer set for amplification of a 1247 bp fragment of the *rec*N gene (supporting information S1 Table and S2 Fig) which is considered to be specific for *S*. *suis* [8, 9].

A small amount of colony material from Columbia sheep blood agar was suspended in molecular grade water (Sigma) to approximately reach McFarland 0.5. In order to lyse the bacteria the suspension was boiled for 10 min and then shock frozen at minus 80°C for 10 min. Five μl of this lysate were added to 20 μl of a master mix containing 1x Phusion HF Buffer, 0.2 mM of each dNTP, 0.2 μM of each primer (0.02 μM for the 16S rRNA gene primers, respectively) and 0.5 U Phusion High-Fidelity DNA Polymerase. The PCR conditions were as follows: an initial denaturation at 94°C for 3 min followed by 30 cycles of denaturation at 94°C for 30 s, primer annealing at 60°C (for grouping PCR) or 58°C (for typing PCR) for 90 s, and extension at 72°C for 45 s, and then a final extension at 72°C for 5 min. The PCR products were visualized after separation in 2% agarose gel by ethidium bromide straining. Results were assigned to serotypes according to the scheme published by Okura et al. [5] if the 16S rRNA gene amplification control was positive. Additionally, *S*. *suis sensu stricto* isolates had to have a 1247 bp fragment representing the *rec*N gene in the grouping PCR.

### Co-agglutination

A subset of isolates was serotyped by co-agglutination with sera detecting serotype 1/2 and serotype 1 to serotype 28, respectively, by courtesy of Hilde Smith at Wageningen University and Research Centre, The Netherlands, as previously described [10]. At least one representative of each of the serotypes detected in our isolate collection by PCR was tested by co-agglutination, in total 26 isolates, as well as all 22 by PCR non-typable isolates from collection B.

### Pathotyping

Extracellular factor (EF), muramidase-released protein (MRP) and suilysin are considered to be virulence associated proteins. We used the detection of the respective genes by PCR to further characterize the isolates. The specific multiplex PCR is described in detail by Silva et al. 2006 including detection of the glutamatdehydrogenase gene (*gdh*) and the arginine deiminase *arc*A gene [11].

### Transmission electron microscopy (TEM)

For morphological analysis of the capsule structure of the genetically and serologically non-typable isolates, samples of early exponential grown bacteria were fixed according to the lysine-acetate-based formaldehyde/glutaraldehyde ruthenium red-osmium (LRR) fixation procedure, as described previously [12] and visualized by transmission electron microscopy (TEM). Thickness and density of the capsule were compared to the serotype 2 reference strain 10, two isogenic mutants without (10Δ*cps*EF) and with impaired capsule (10Δ*ccp*A) expression and a complemented *ccp*A mutant (c10Δ*ccp*A) prepared with the same method and described in a previous study [13].

### Statistical analysis

Statistical analysis was done with SAS Enterprise Guide 7.1 using Fisher´s exact test. The significance levels were as follows: 0.01<p≤0.05, significant, indicated by *; 0.001<p≤0.01, very significant, indicated by **; p≤0.001, highly significant, indicated by ***.

## Results

In all 711 isolates from collection A (1996–2004) and B (2015–2016) investigated in this study, including all non-*cps*-typable isolates, *gdh* and *rec*N could be detected by the two PCR assays implemented. This is in accordance with our finding that none of the isolates was assigned to serotype 20, 22, 26, 32, 33 or 34 by PCR, which are no longer considered to belong to the species *S*. *suis* but rather to other *Streptococcus* species [8].

### Frequency of serotypes in collection A vs. collection B

In collection A overall 19 different *cps* types were found among the 189 isolates (Table 1). The most frequent *cps* type was serotype-2/serotype-1/2, which added up to 26.5% of the isolates. Serotype 9, serotype 4 and serotype 7 accounted for 11.1%, 10%, and 9% of the isolates, respectively, and serotype 3, serotype 5 and serotype-1/serotype-14 for 7.4%, 6.4%, and 5.3%, respectively. Twelve other *cps* types were isolated less frequently (< 3%). A proportion of 9% of the isolates was genetically non-typable.

**Table 1:** Number [percentage] of isolates for each *cps* type in collection A and collection B, respectively.

*cps* type	Collection A		Collection B	
	n	[%]	n	[%]
1 and 14	10	5,3	25	4,8
2 and 1/2	50	26,5	108	20,7
3	14	7,4	26	5
4	19	10,0	54	10,3
5	12	6,4	14	2,7
6	0	0	0	0
7	17	9	64	12,3
8	4	2,1	28	5,4
9	21	11,1	88	16,9
10	0	0	4	0,8
11	2	1,0	5	1
12	3	1,6	0	0
13	1	0,5	2	0,4
15	5	2,7	7	1,3
16	1	0,5	9	1,7
17	0	0	1	0,2
18	0	0	10	1,9
19	0	0	7	1,3
20	0	0	0	0
21	4	2,1	6	1,2
22	0	0	0	0
23	1	0,5	4	0,8
24	1	0,5	3	0,6
25	0	0	0	0
26	0	0	0	0
27	0	0	0	0
28	0	0	10	1,9
29	1	0,5	10	1,9
30	1	0,5	2	0,4
31	5	2,7	13	2,5
32	0	0	0	0
33	0	0	0	0
34	0	0	0	0
nt^a^	17	9	22	4,2
∑	189	100	522	100

^a^ nt: genetically non-typable

The more recent collection B encompassed a total of 23 different *cps* types among 522 isolates (Table 1). As in collection A, serotype-2/serotype-1/2 was the most frequent serotype (20.7%). Serotype 9, 7, 4 and 8 were isolated frequently as well (16.9%, 12.3%, 10.3% and 5.4%, respectively). Only 4.2% of the isolates were non-typable by PCR.

Differences in *cps* type prevalence between the two collections irrespective of the origin of isolation were not considered since invasive, lung and carrier isolates were unequally distributed in the two collections. However, when the isolates were grouped based on the anamnestic information into invasive, pulmonary and carrier isolates, such differences could be analyzed (see below and supporting information S2 Table).

#### Invasive isolates

**Fig 1:**
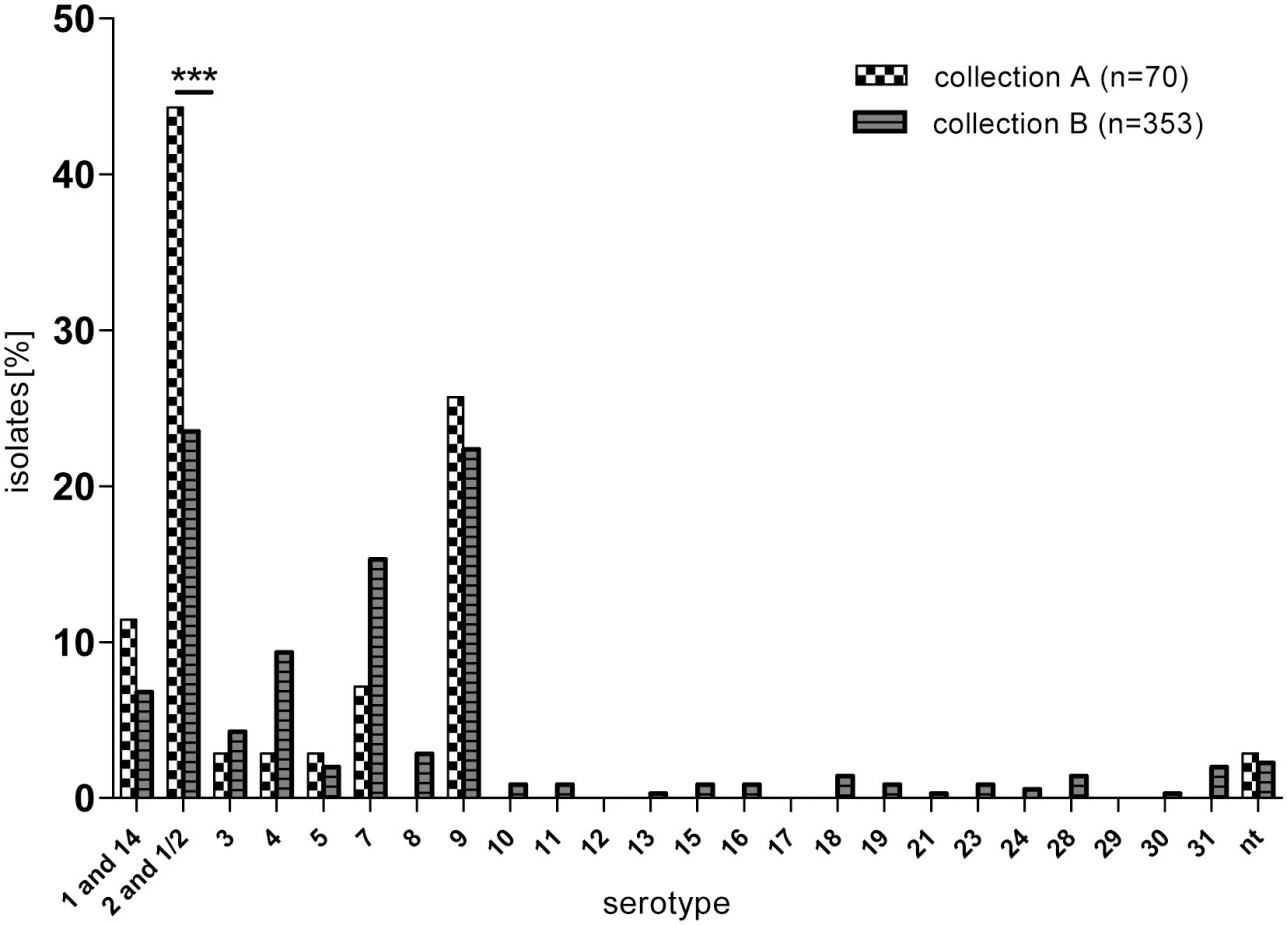
Frequency of different serotypes based on *cps* typing among *S*. *suis* invasive isolates collected between 1996–2004 (A) and 2015–2016 (B), respectively. nt: genetically non-typable; ***p≤0.001 highly significant.

Among the invasive group (Fig 1 and supporting information S2 Table) serotype-2/serotype-1/2 was most common in both collections. However, the proportion of these isolates was 44.3% in collection A and only 23.5% in collection B, revealing a highly significant difference (p = 0.0006) between the collections. In contrast, serotype 9 was equally represented in both collections among invasive isolates (25.7% and 22.4%, respectively). The frequency of isolation of serotype 4 and 7 tended to increase over the years but this was just below statistical significance. Serotypes other than serotype-1/serotype-14, serotype-2/serotype-1/2, serotype 3 to serotype 5 and serotype 7 to serotype 9 were not, or only rarely identified (<2% each), as were genetically non-typable isolates (collection A: 2.9%; collection B: 2.3%).

Within the group of invasive isolates, the shift of *cps* types between the two collections was especially pronounced when confining to CNS isolates only (supporting information S3 Table). Among CNS isolates in collection B there was a highly significant lower proportion of serotype-2/serotype-1/2 isolates (52.1% in collection A vs. 23.2% in collection B; p = 0.0002) and a significant lower frequency of serotype-1/serotype-14 (8.3% in collection A vs. 1.7% in collection B; p = 0.0316). On the other hand, isolates belonging to serotypes 4, 7 and 8 tended to be more frequently found in collection B than in collection A, but this was statistically not significant.

**Fig 2:**
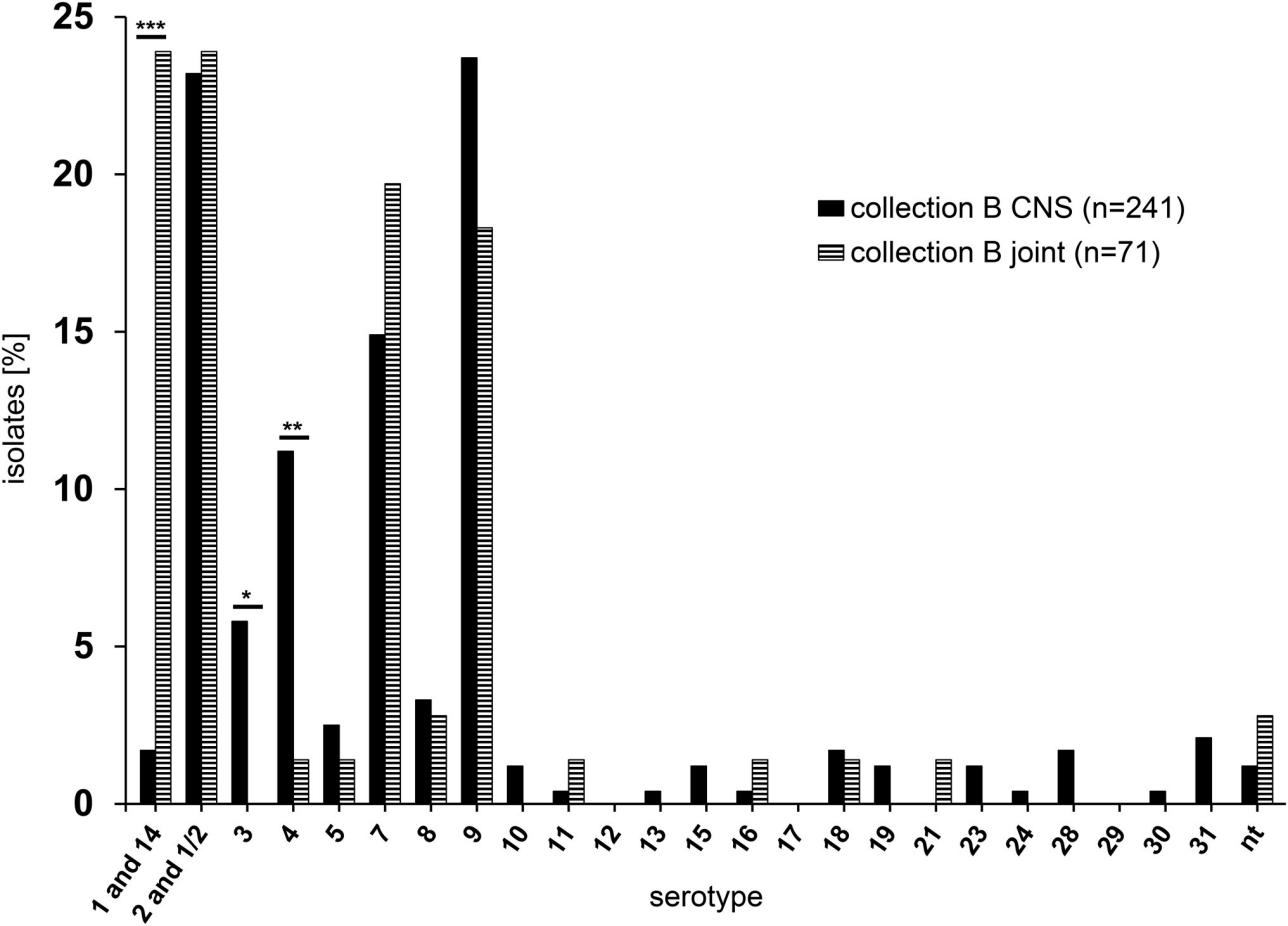
Frequency of different serotypes based on *cps* typing in CNS vs. joint isolates among invasive *S*. *suis* isolates in collection B (2015–2016). nt: genetically non-typable; * 0.01<p≤0.05 significant; ** 0.001<p≤0.01 very significant; *** p≤0.001 highly significant.

Notably, 27 of the 33 invasive serotype 4 isolates in collection B originated from the CNS (11.2% of all CNS isolates) and only one had been isolated from joints (1.4% of all isolates from joints; Fig 2). Thus, in collection B isolates from the CNS significantly more often belonged to serotype 4 than isolates from joints (p = 0.0083). This was also found for serotype 3 encompassing 5.8% of the isolates from the CNS and none from joints (p = 0.0454).

Conversely, in collection B isolates from joints highly significantly more often belonged to serotype-1/serotype-14 when compared to isolates from the CNS (23.9% of all isolates from joints vs. 1.7% of all isolates from the CNS; p<0.0001).

In both collections altogether only 3 of 289 isolates from the CNS (1.0%) and only 4 of 83 isolates from joints (4.8%) were non-typable by PCR.

#### Respiratory isolates

**Fig 3:**
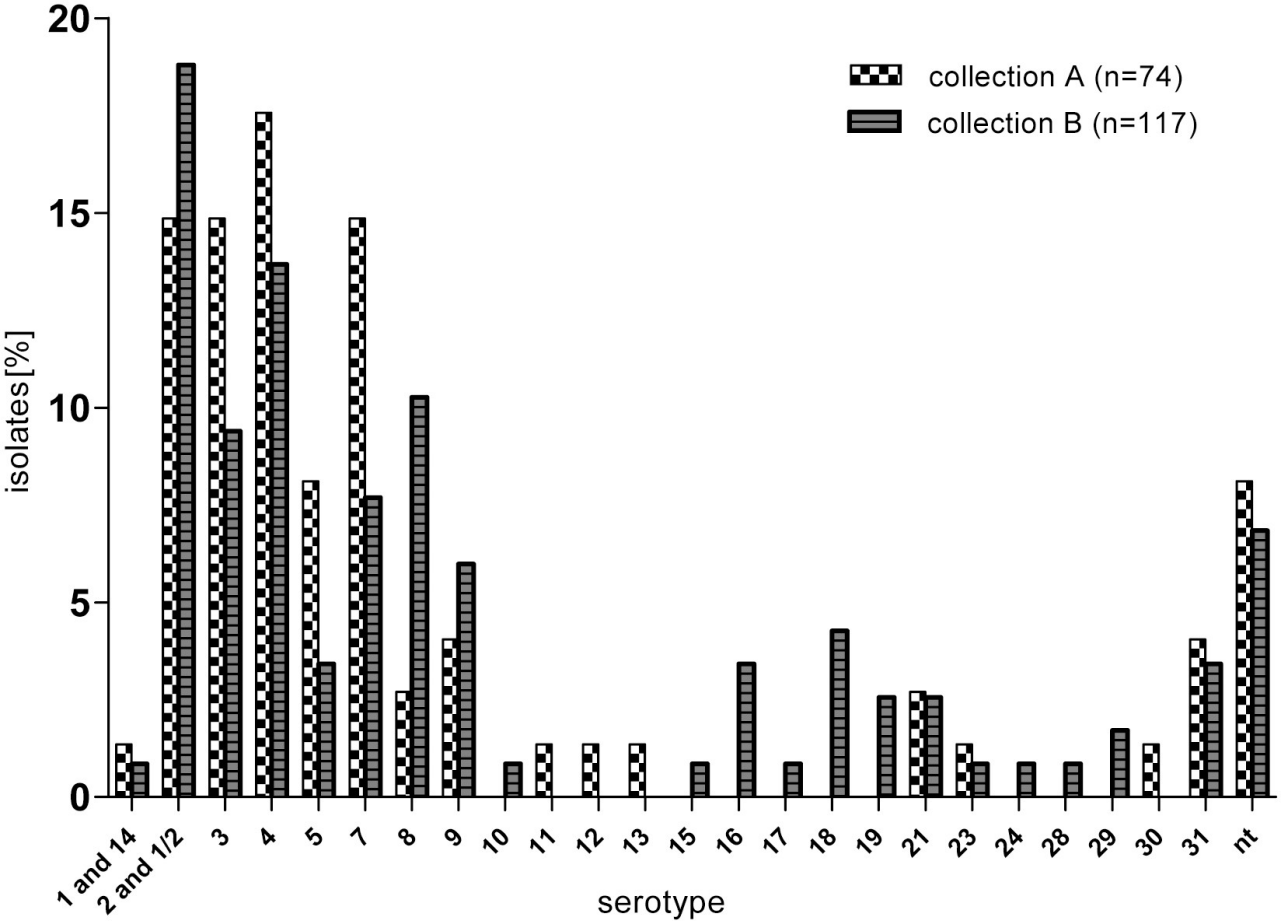
Frequency of different serotypes based on *cps* typing among *S*. *suis* pulmonary isolates collected between 1996–2004 (A) and 2015–2016 (B), respectively. nt: genetically non-typable.

Among pulmonary isolates (Fig 3 and supporting information S2 Table) in both collections, the variety of detected *cps* types was larger than among invasive isolates. Almost 14% (13.7% collection A) and 23.4% (collection B) of the isolates belonged to either of the serotypes 10 to 31, with serotype 31 being as frequent as serotype 9 in collection A (4.0%). However, most pulmonary isolates belonged to serotype-1/serotype-14 to serotype 5 and serotype 7 to serotype 9 (78.2% in collection A; 69.8% in collection B). Genetically non-typable pulmonary isolates were more frequently found than among invasive isolates in both collections (8.1% and 6.8% vs. 2.9% and 2.3%, respectively).

Overall, in collection A serotype 4 was the most frequent serotype in pulmonary isolates (17.6%) followed by serotype-2/serotype-1/2, serotype 3 and serotype 7 (14.9% each). In collection B serotype-2/serotype-1/2 was more frequent than serotype 4 (18.8% vs. 13.7%) followed by serotype 8 and 3 (10.3% and 9.4%). However, differences in *cps* type distribution between the two collections were not statistically significant for pulmonary isolates.

#### Carrier isolates

**Fig 4:**
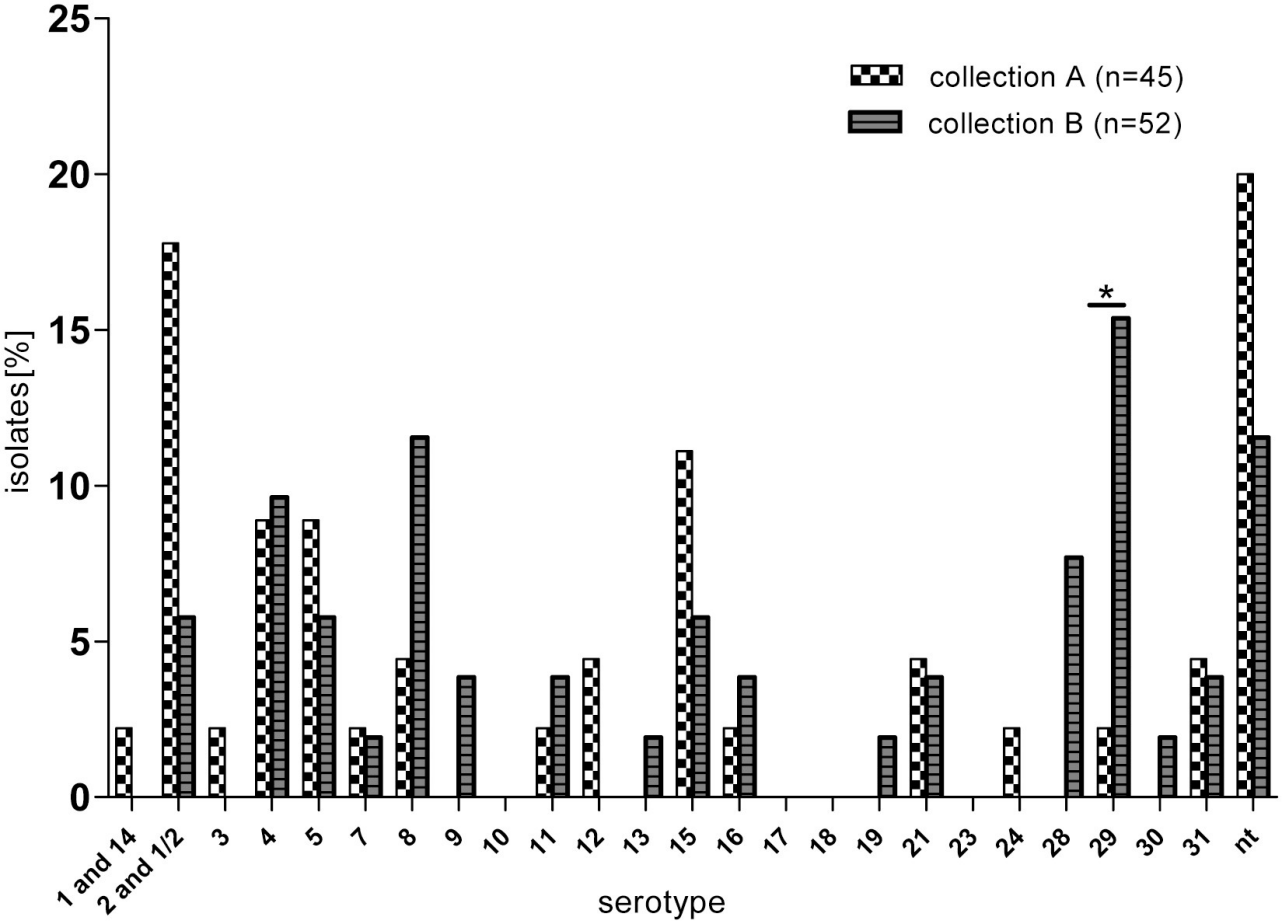
Frequency of different serotypes based on *cps* typing among *S*. *suis* carrier isolates collected between 1996–2004 (A) and 2015–2016 (B), respectively. nt: genetically non-typable; * 0.01<p≤0.05 significant.

Among carrier isolates (Fig 4 and supporting information S2 Table) a very large variety of different *cps* types was detected including a marked proportion of genetically non-typable isolates of 20% of the isolates of collection A and 11.5% of collection B, respectively. In collection A serotype-2/serotype-1/2 and in collection B serotype 29 was most frequent. The difference in frequency of serotype 29 between both collections (A: 2.2%; B: 15.4%) was statistically significant (p = 0.0347). However, the small number of carrier isolates (45 in collection A and 52 in collection B) limits interpretation of this subset.

Molecular and phenotypic serotyping was in accordance for 22 of the 26 isolates representatively selected from those with a distinct *cps* type assigned by PCR (supporting information see S4 Table). Two isolates could not be phenotyped because of auto-agglutination, one serotype 15 isolate by *cps* typing and one serotype 28 isolate by *cps* typing. One by *cps* typing serotype 10 isolate repeatedly reacted only with an antiserum to serotype 9 and another isolate of serotype 10 reacted with an antiserum to serotype 10, 9, 21 and 22 (poly-agglutination). The serotype-specific *wzy* gene of both isolates was sequenced and showed 100% identity with the sequence of *S*. *suis* strain 4417, a serotype 10 isolate, (GenBank accesion number JX986799) using Megablast with default settings (http://blast.ncbi.nlm.nih.gov/Blast.cgi) and the database Nucleotide collection nr/nt. Besides, none of the two isolates produced a signal with a different *cps* typing PCR based on a different primer set for serotype 9 detection [11].

In addition to the 26 isolates representing distinct molecular *cps* types, all 22 isolates of collection B, which were non-typable by PCR, could not be assigned to a specific serotype with co-agglutination either.

### Pathotyping

**Fig 5:**
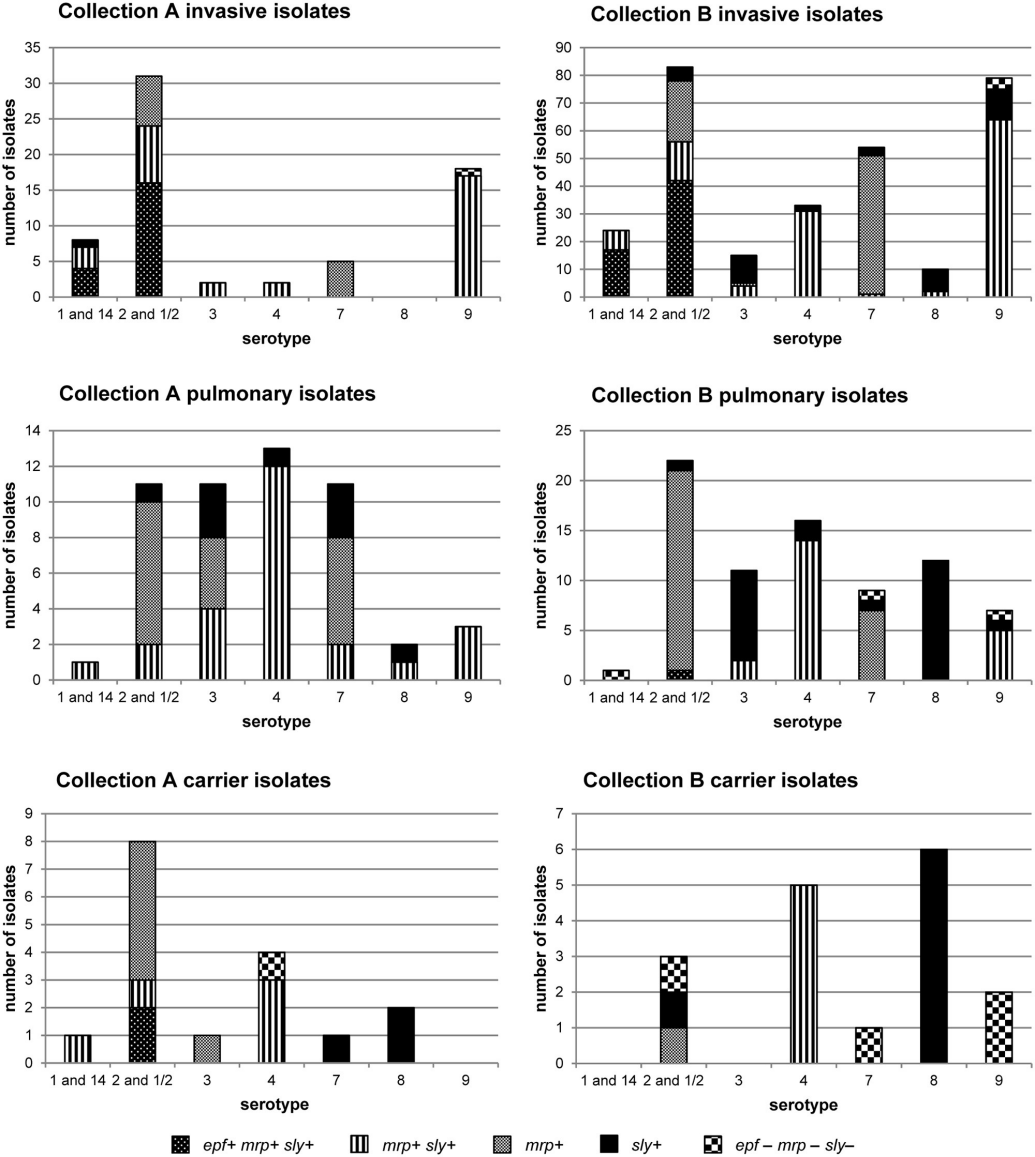
Pathotypes of selected serotypes based on *cps* typing of *S*. *suis* from collection A (1996–2004) and collection B (2015–2016). Number of isolates with/without genes for extracellular factor (*epf*), muramidase-released protein (*mrp*) and suilysin (*sly*) alone or in combination.

The *epf-*gene was only detected in serotype-1/serotype-14 and serotype-2/serotype-1/2 isolates and always in combination with *mrp* and *sly* (Fig 5, supporting information S5 Table). This genotype was seen in invasive isolates (51.3% in collection A; 55.1% in collection B), in two carrier serotype-2/serotype-1/2 isolates in collection A and in one pulmonary serotype-2/serotype-1/2 isolate in collection B. Association of *epf* with invasive isolates was statistically significant in collection B (p<0.001). In lung isolates serotype-2/serotype-1/2 was mainly associated with *mrp* (72.7% in collection A; 91% in collection B). Invasive and pulmonary isolates of serotype 4 in both collections carried predominantly *mrp* and *sly* (93.3% and 91.8% of the isolates of collection A and B, respectively). This was also true for serotype 9 isolates from systemic disease or lungs (95.2% and 80.2%% of the isolates in the two collections, respectively). In collection B serotype 7 isolates mostly carried *mrp* (89.1%) and serotype 3 and serotype 8 isolates carried *sly* irrespective of the clinical background (73.1% and 91% of the isolates of serotype 3 and 8, respectively). Similarly, 68.8% serotype 7 isolates in collection A also were positive for *mrp* only. However, serotype 3 isolates in this collection harbored *mrp* and *sly* (46.2%), *mrp* only (30.8%) and less frequently *sly* alone (23.1%). Across all serotypes *mrp* or *sly* were significantly less frequently found in carrier isolates than in lung or invasive isolates (p<0.001) and more lung isolates possessed *sly* than invasive isolates (p = 0.004).

### Analysis of capsule expression by TEM

TEM analysis revealed that none of the 22 isolates that were non-typable by molecular methods and co-agglutination expressed a capsule comparable to that of a serotype 2 control strain (score ++++; see supporting information S3 Fig and S6 Table). However, the capsule score of two non-typable isolates (2016/04646/02/05 and 2016/04144/09/09) was similar to a complemented mutant strain (c10Δ*ccp*A) in the serotype 2 control strain background (score +++). Moreover, six isolates seemed to be covered by capsular material less than this complemented mutant but still discernable (score ++) and distinct from the respective mutant strain 10Δ*ccp*A with an impaired capsule expression (score +). Only isolate 2016/03188/04/17 lacked any capsular material (-), whereas 13 isolates showed varying degrees of thin material covering the surface indicating insufficient or defective capsular expression (+) like in strain 10Δ*ccp*A.

## Discussion

The capsule of *S*. *suis* is a major virulence factor for several serotypes during certain stages of the infection [12, 14–20] It is also an important target for initial epidemiological characterization of this pathogen by serotyping. Until recently, serotyping required availability of rabbit hyperimmune sera. Furthermore, it was hampered by some methodological challenges, such as co-agglutination (auto-, poly- or cross-agglutination) and subjectivity of interpretation of results.

From several published PCR assays designed for detecting important [11], many, or all serotypes of *S*. *suis* [5, 21, 22], we selected the method described by Okura et al. in 2014 [5] to examine two collections of *S*. *suis* isolates from pigs in Germany which were compiled approximately 10 years apart. This two-step multiplex PCR detects all 35 serotypes including those no longer assigned to the species *S*. *suis*. However, it cannot distinguish serotype 1 and serotype 14 or serotype 2 and serotype 1/2. To ensure the identification of isolates as *S*. *suis*, we incorporated a primer pair detecting *rec*N which is now considered to be a specific and reliable identification assay for *S*. *suis* isolates [23, 24]. All field isolates included in this study were positive for *rec*N and consequently none of them did belong to serotype 20, 22, 26, 32, 33 or 34, which are now considered to belong to other *Streptococcus* species [8].

When comparing a representative isolate from our collection of each of the serotypes detected by *cps* typing to co-agglutination results, there was an overall very good agreement between both methods. Three isolates could not be identified by co-agglutination because of auto- and poly-agglutination, respectively. One isolate identified as serotype 10 by *cps* typing reacted with serotype 9 antiserum even though the sequence of the *wzy* gene matched exactly to the sequence of the *wzy* gene of another serotype 10 isolate deposited at GenBank. We considered this result a cross-agglutination phenomenon between the serotype 10 capsule and the serotype 9 serum, which has been described before for different serotypes [6, 25–28].

Notably, 22 *S*. *suis* isolates from collection B could not be assigned to any known serotype, neither by PCR nor by co-agglutination. Thirteen isolates reacted with none of the sera, two showed polyagglutination and seven autoagglutination. However, TEM revealed that two isolates displayed a capsule, which was similar in thickness and density as compared to a complemented serotype 2 capsule-defective mutant. Only one of the 22 isolates was clearly non-encapsulated, whereas all other isolates showed varying amounts and densities of capsular material. Therefore, we assume that at least some of these isolates may belong to new, yet undescribed serotypes including those characterized by novel *cps* loci (NCL) [29–31]. For other isolates, there may be mutations in the primer annealing sites, which interfere with successful amplification. Since none of the isolates could be serotyped by co-agglutination using sera for detecting serotype 1/2 and 1 to 28 either, single nucleotide polymorphisms (SNPs) in the primer sites might be only an explanation for non-typability in *rec*N-positive isolates of serotypes 29 to 31. Notably, five of the eight invasive isolates that could not be typed, neither genetically nor serologically, showed a moderate capsule expression (s. supporting information S6 Table), that might contribute to their virulence.

For a detailed analysis of the frequency of individual serotypes we subdivided the two isolate collections from 1996–2004 (A) and from 2015–2016 (B) into invasive, respiratory and carrier isolates, since the respective collections were assembled differently. In collection A one third of the isolates were from systemic infection while in collection B two thirds of the isolates had this anamnestic background. For the invasive isolates we observed a highly significant lower number of serotype-2/serotype-1/2 isolates in the more recent collection B. Since more than 10 years vaccination for streptococcal infections with farm-specific autogenous vaccines has been widely practiced in Germany. Several authors have shown that vaccination with serotype-2/serotype-1/2 bacterins can be quite effective [32–34] Thus, vaccination with serotype serotype-2/serotype-1/2 bacterins may have caused the displacement of this type by other serotypes invading the ecological niche over the last ten years. This phenomenon is well known in human populations vaccinated with polyvalent *S*. *pneumonia* vaccines resulting in an increase of non-vaccine serotypes (NVTs) in those populations [35]. However, it may also be possible that the change is due to serotype switching as it has been described for *S*. *pneumoniae* [36–38] and discussed for Dutch *S*. *suis* isolates possessing a type-I restriction modification (R-M) locus [39]. The detection rate for the second most common serotype, serotype 9, seems to be stable over the last ten years, at least in Germany. Serotype 9 isolates are also used as autogenous vaccine, but less successfully, which is in accordance with experimental studies [33]. Dekker et al. showed that vaccination with a serotype 9 strain at three and five weeks of age did not reduce colonization and transmission after inoculation with a homologous strain two weeks later [40]. In the field it remains to be elucidated whether vaccination may affect spreading of serotype 9 strains to some extent depending on vaccination scheme and other factors like choice of adjuvant.

Serotype-1/serotype-14 accounted only for 6.8%% of the invasive isolates of collection B, but most of these isolates were from joints (17 isolates from joints, 4 from CNS, 3 from various other locations). This difference in serotype prevalence between isolates from joints and CNS was statistically highly significant in collection B, and it was also discernable in collection A. However, total numbers of isolates were too small in collection A to assess statistical significance. While serotype-1/serotype-14 isolates seemed to have a tissue tropism to joints, serotype 4 and serotype 3 isolates were overrepresented in CNS samples in collection B, which was statistically significant, too.

There were no statistically valid changes in the frequency and distribution of *S*. *suis* serotypes between the two collections of lung isolates. This included serotype-2/serotype-1/2 isolates, which seemed to be unaffected by vaccination with bacterins from invasive serotype-2/serotype-1/2 isolates. The two *S*. *suis* populations, invasive serotype-2/serotype-1/2 and pulmonary isolates of this serotype, appeared to be different as can be deduced from differences in profiles of virulence-associated genes in this serotype depending on the origin of isolation of the isolates. While in both collections 84.8% of the pulmonary isolates of serotype-2/serotype-1/2 harbored *mrp* only, 70.2% of the invasive isolates of this serotype carried both *mrp* and *sly*, and 50.9% of them additionally *epf*.

In contrast to our previous study [11], there was no indication of an increased tropism of serotype 7 isolates for the lung. Actually, in collection A, which is almost identical to that used in our previous study, serotype 4 was more frequently detected in the lung than serotype 7. However, this serotype had not been included in the previously used PCR, and these isolates consequently “vanished” in the genetically non-typable group. Notably, the clinical significance of the lung isolates is difficult to evaluate. The broader range of detected serotypes and the larger amount of genetically non-typable isolates in this group compared to the invasive isolates may indicate a secondary role in lung disease or such isolates may even represent “carrier” isolates.

While serotype-1/serotype-14, serotype-2/serotype-1/2, serotype 3 to 5 and serotype 7 to 9 predominated especially among invasive isolates, as well as among lung isolates, in carrier isolates a broad range of different serotypes was identified with serotype 29 being especially prominent in collection B. A recent study on a collection of 127 carrier isolates from the UK and 223 isolates from China described serotype 16 as the most frequent type while serotype 29 was only found once in the UK and 12 times in the Chinese collection [41].

Still, some of the potentially more virulent serotypes found in invasive isolates were also present in carrier animals, which hence constitute a reservoir and a source of infection for naïve animals. Two serotype-2/serotype-1/2 isolates carrying *epf*, *mrp* and *sly* were obtained from carrier animals from collection A and one originated from the lung in collection B. However, as mentioned before, for lung isolates an assignment to respiratory disease is sometimes difficult, i.e. this *epf* positive isolate may originate from a systemic infection, which has spread from a respiratory infection. Invasive and lung isolates of serotypes 4 and 9 in both collections and of serotypes 3, 7 and 8 in collection B were similar in pathotype. Factors other than the ones tested for in our PCR may contribute to higher invasiveness of some isolates of these serotypes and distinguish the two populations. The genes for all three virulence-associated factors, EF, MRP and suilysin, were significantly less frequently detected in carrier isolates than in clinical isolates, which supports their association with virulence. EF seems to be strongly correlated to invasiveness of isolates in serotype-1/serotype-14 and also serotype-2/serotype-1/2, whereas suilysin was most frequently associated with lung isolates. The relevance of the latter observation for pathogenicity remains to be elucidated.

Taken together, molecular characterization of *S*. *suis* isolates between 1996 and 2016 with respect to serotype and pathotype revealed some interesting changes over the years and distinctions for isolates from different clinical background. With respect to the notion that humans may be especially vulnerable to zoonotic infection with serotype 2 isolates, the decrease of this serotype among more recently collected isolates from pigs is good news. However, other and possibly new serotypes may exploit this ecological niche in pigs and possibly in humans as well. Finally, the somewhat neglected serologically and genetically non-typable isolates seem to express various amounts of capsular polysaccharides pointing towards the possibility of new serotypes.

## Supporting information

S1 Fig Geographic origin of samples from which *S*. *suis* was isolated. A: collection A (1996–2004); B: collection B (2015–2016) Legend: number of isolates Note: high numbers of isolates correspond to areas with dense pig population The map was generated with our data using the software package “Das Postleitzahlen-Diagramm 4.0” by Klaus Wessiepe (http://www.Klaus-Wessiepe.de) licensed for „Institut für Mikrobiologie, Tierrztliche Hochschule Hannover“, 2007.
(PDF)

S2 Fig Specificity of *rec*N primers. Lane 1 100 bp DNA Ladder (NEB); lane 2 *S*. *suis* ATCC 43765 ^T^; lane 3 *S*. *suis* serotype 20 strain 86–5192; lane 4 *S*. *suis* serotype 22 strain 88–1861; lane 5 *S*. *suis* serotype 26 strain 89-4109-1; lane 6 *S*. *suis* serotype 32 strain EA 1172.91; lane 7 *S*. *suis* serotype 33 strain EA 1832.92; lane 8 *S*. *suis* serotype 34 strain 92–2742; lane 9 *Streptococcus porcinus* ATCC 12391; lane 10 *Streptococcus dysgalactiae* NCFB 2043; lane 11 *Enterococcus faecalis* ATCC 29212; lane 12 *Enterococcus faecium* ATCC 6057; lane 13 *Aerococcus viridans* ATCC 11563^T^; lane 14 *Lactococcus lactis* IMET 13300; lane 15 *Leuconostoc carnosum* ATCC 49367^T^; lane 16 A. dest.; lane 17 100 bp DNA Ladder (NEB).
(PDF)

S3 Fig TEM images of serologically and genetically non-typable *S*. *suis* isolates. TEM images of non-typable S. suis isolates representing an isolate with a well expressed capsule (A), a moderately well expressed capsule (B and C), a defective capsule (D), a very defective capsule (E) and no capsule (F). For details s. supporting information S6 Table. The scale corresponds to 0.2 μm.
(PDF)

S1 Table Primers for *rec*N amplification integrated into the two-step multiplex PCR by Okura et al. (J Clin Microbiol. 2014;52(5):1714–9.). (PDF)

S2 Table Serotypes of German *S*. *suis* isolates based on *cps* typing in collection A (1996–2004) and collection B (2015–2016), respectively, grouped by disease status. ^a^ nt: genetically non-typable by PCR.
(PDF)

S3 Table Serotypes of German invasive *S*. *suis* isolates based on *cps* typing in collection A (1996–2004) and collection B (2015–2016), respectively, grouped by origin of isolation. ^a^ nt: genetically non-typable by PCR. ^b^ CNS: central nervous system. c Other: internal organs other than lungs. * 0.01<p≤0.05 significant difference between collection A and B or ^†^ within collection B between CNS and joint isolates. ^††^ 0.001<p≤0.01 very significant difference within collection B between CNS and joint isolates *** p≤0.001 highly significant difference between collection A and B or ^†††^ within collection B between CNS and joint isolates.
(PDF)

S4 Table Comparison of molecular and phenotypic serotyping for a representative selection of *S*. *suis* isolates. ^a^ origin 1 = invasive; 2 = pulmonary; 3 = carrier. ^b^ courtesy of Dr. Hilde Smith, Central Veterinary Institute of Wageningen University Research; with sera for serotype 1/2 and 1–28. ^c^ no agglutination with serum 10 in repeated tests. ^d^ sequencing of the PCR-fragment showed 100%-identity with the *wzy* gene of another serotype 10 isolate in GenBank (https://www.ncbi.nlm.nih.gov/gene).
(PDF)

S5 Table Pathotype of selected serotypes (ST) based on *cps* typing of invasive, pulmonary and carrier *S*. *suis* isolates from collection A (1996–2004) and collection B (2015–2016). Number of isolates with/without genes for extracellular factor (*epf*), muramidase-released protein (*mrp*) and suilysin (*sly*) alone or in combination.
(PDF)

S6 Table Characterization of the capsule of serologically and genetically non-typable *S*. *suis* isolates from collection B as revealed from TEM images. ^a^ according to WILLENBORG et al. (2011). ^b^ origin 1 = invasive; 2 = pulmonary; 3 = carrier. ^c^ density:++++ extremely dense / +++ very dense / ++ moderately dense / + slightly dense / (+) very slightly dense /—no fuzzy material. ^d^ thickness:++++ ≥ 120 nm / +++ 90–119 nm / ++ 60–89 nm / + 30–59 nm / (+) ≤ 29 nm /—no fuzzy material. ^e^ score: average of density and thickness ++++ very well expressed capsule / +++ well expressed capsule / ++ moderately expressed capsule / + defective capsule / (+) very defective capsule /—no capsule.
(PDF)
