# Supplementary material for: Molecular typing of Streptococcus suis strains isolated from diseased and healthy pigs between 1996-2016
Source: PLoS One. 2019 Jan 17;14(1):e0210801. doi: 10.1371/journal.pone.0210801 (PMC6336254; doi:10.1371/journal.pone.0210801)
Supplement: S1 Fig — A: collection A (1996–2004); B: collection B (2015–2016) Legend: number of isolates Note: high numbers of isolates correspond to areas with dense pig population The map was generated with our data using the software package “Das Postleitzahlen-Diagramm 4.0” by Klaus Wessiepe (http://www.Klaus-Wessiepe.de) licensed for „Institut für Mikrobiologie, Tierrztliche Hochschule Hannover“, 2007. (PDF) [file pone.0210801.s001.pdf]

S1 Fig. Geographic origin of samples from which *S. suis* was isolated

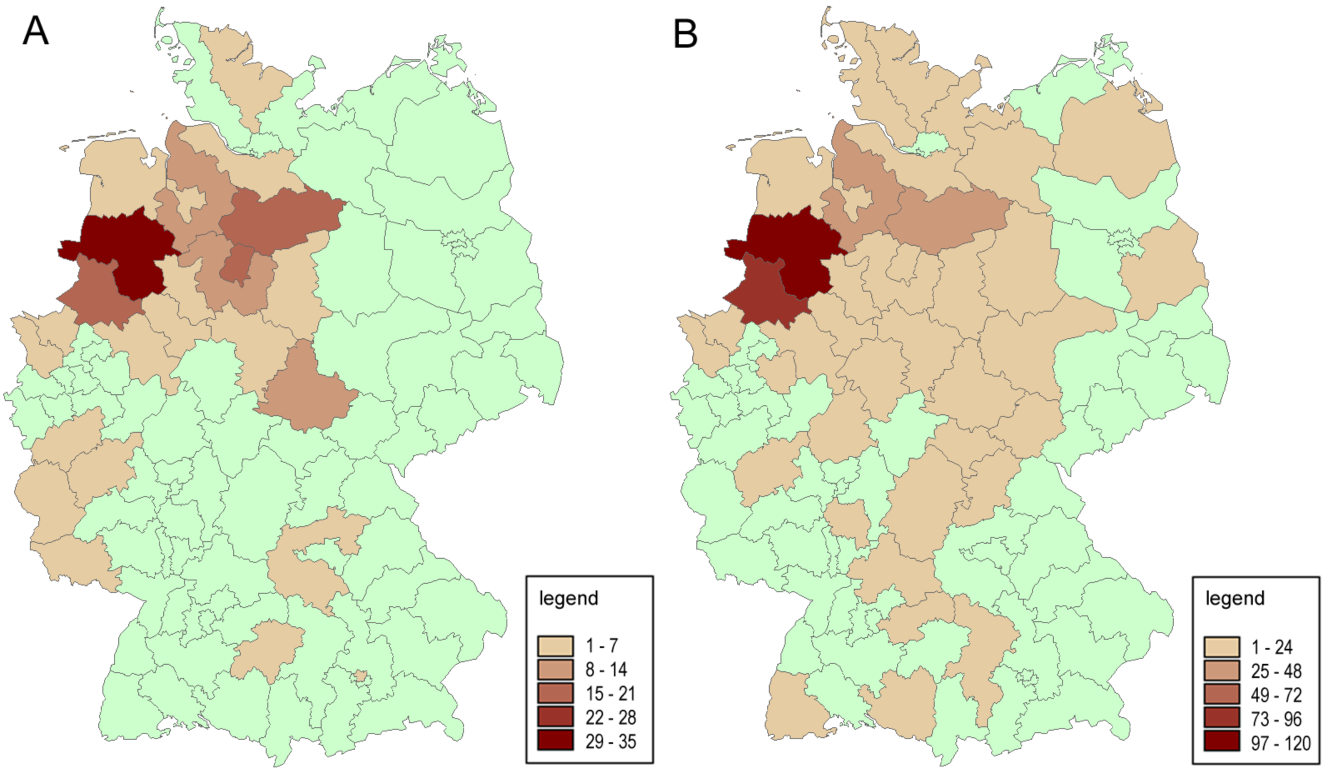

A: collection A (1996-2004)
B: collection B (2015-2016)
Legend: number of isolates

Note: high numbers of isolates correspond to areas with dense pig population

The map was generated with our data using the software package “Das Postleitzahlen-Diagramm 4.0” by Klaus Wessiepe (www.Klaus-Wessiepe.de) licensed for „Institut für Mikrobiologie, Tierärztliche Hochschule Hannover“, 2007.
