# Supplementary material for: Molecular typing of Streptococcus suis strains isolated from diseased and healthy pigs between 1996-2016
Source: PLoS One. 2019 Jan 17;14(1):e0210801. doi: 10.1371/journal.pone.0210801 (PMC6336254; doi:10.1371/journal.pone.0210801)
Supplement: S2 Fig — Lane 1 100 bp DNA Ladder (NEB); lane 2 S. suis ATCC 43765 T; lane 3 S. suis serotype 20 strain 86–5192; lane 4 S. suis serotype 22 strain 88–1861; lane 5 S. suis serotype 26 strain 89-4109-1; lane 6 S. suis serotype 32 strain EA 1172.91; lane 7 S. suis serotype 33 strain EA 1832.92; lane 8 S. suis serotype 34 strain 92–2742; lane 9 Streptococcus porcinus ATCC 12391; lane 10 Streptococcus dysgalactiae NCFB 2043; lane 11 Enterococcus faecalis ATCC 29212; lane 12 Enterococcus faecium ATCC 6057; lane 13 Aerococcus viridans ATCC 11563T; lane 14 Lactococcus lactis IMET 13300; lane 15 Leuconostoc carnosum ATCC 49367T; lane 16 A. dest.; lane 17 100 bp DNA Ladder (NEB). (PDF) [file pone.0210801.s002.pdf]

## S2 Fig. Specificity of *rec*N primers

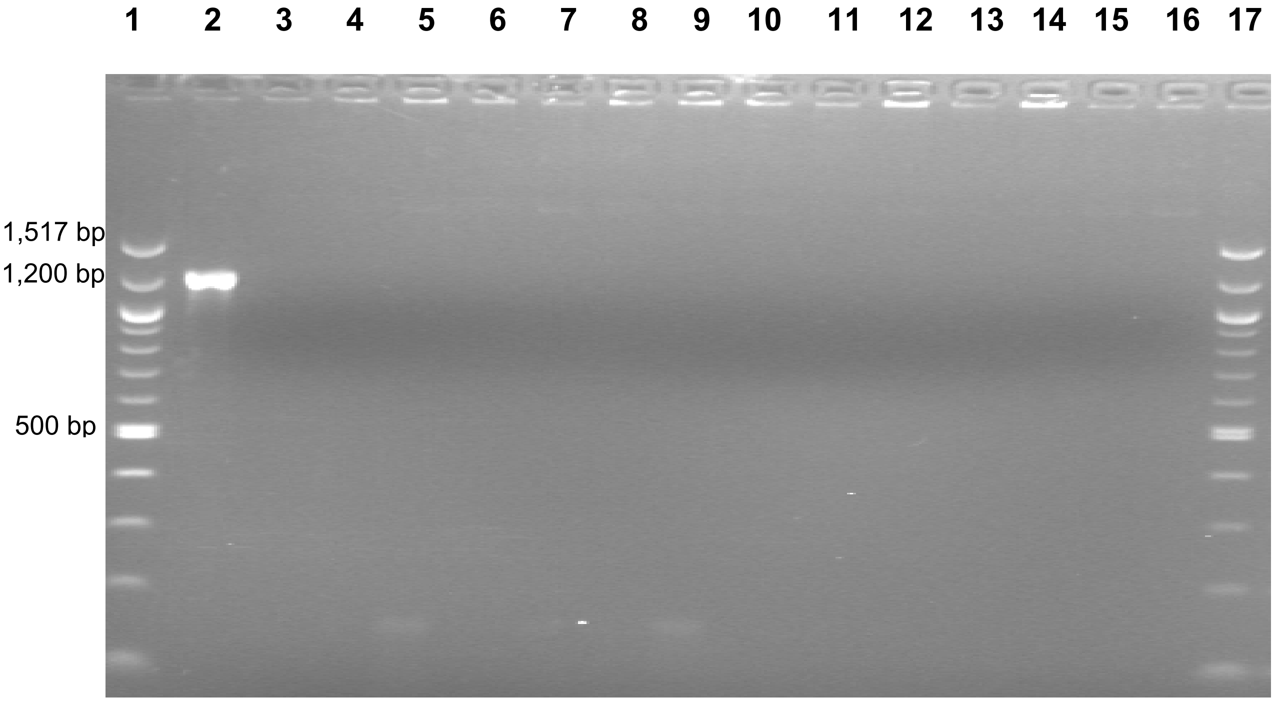


Lane 1 100 bp DNA Ladder (NEB); lane 2 *S. suis* ATCC 43765 $^{T}$; lane 3 *S. suis* serotype 20 strain 86-5192; lane 4 *S. suis* serotype 22 strain 88-1861; lane 5 *S. suis* serotype 26 strain 89-4109-1; lane 6 *S. suis* serotype 32 strain EA 1172.91; lane 7 *S. suis* serotype 33 strain EA 1832.92; lane 8 *S. suis* serotype 34 strain 92-2742; lane 9 *Streptococcus porcinus* ATCC 12391; lane 10 *Streptococcus dysgalactiae* NCFB 2043; lane 11 *Enterococcus faecalis* ATCC 29212; lane 12 *Enterococcus faecium* ATCC 6057; lane 13 *Aerococcus viridans* ATCC 11563$^{T}$; lane 14 *Lactococcus lactis* IMET 13300; lane 15 *Leuconostoc carnosum* ATCC 49367$^{T}$; lane 16 A. dest.; lane 17 100 bp DNA Ladder (NEB)
