# Supplementary material for: Molecular typing of Streptococcus suis strains isolated from diseased and healthy pigs between 1996-2016
Source: PLoS One. 2019 Jan 17;14(1):e0210801. doi: 10.1371/journal.pone.0210801 (PMC6336254; doi:10.1371/journal.pone.0210801)
Supplement: S3 Fig — TEM images of non-typable S. suis isolates representing an isolate with a well expressed capsule (A), a moderately well expressed capsule (B and C), a defective capsule (D), a very defective capsule (E) and no capsule (F). For details s. supporting information S6 Table. The scale corresponds to 0.2 μm. (PDF) [file pone.0210801.s003.pdf]

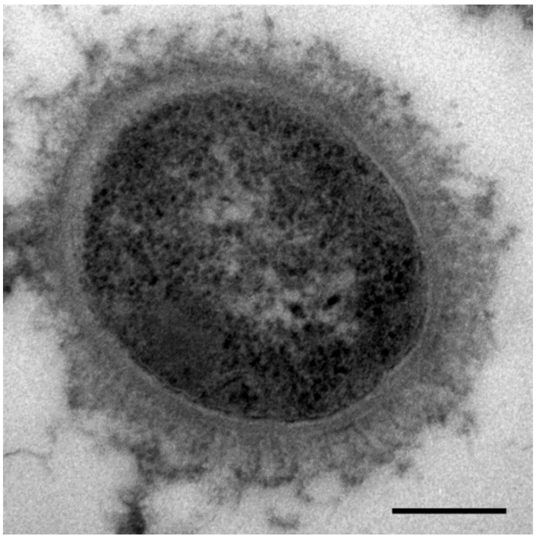

A 2016/04646/02/05

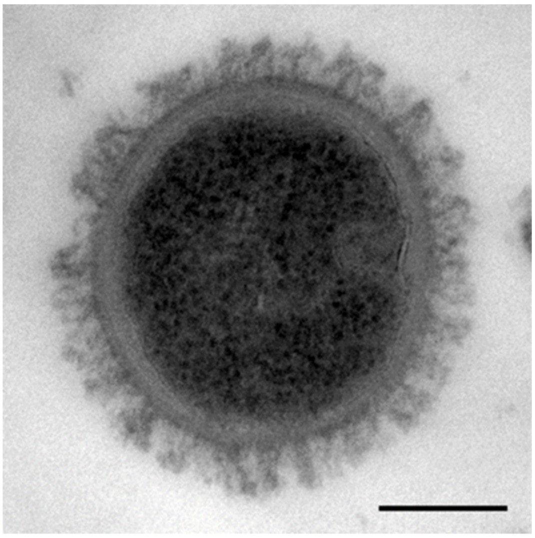

B 2016/01183/05/05

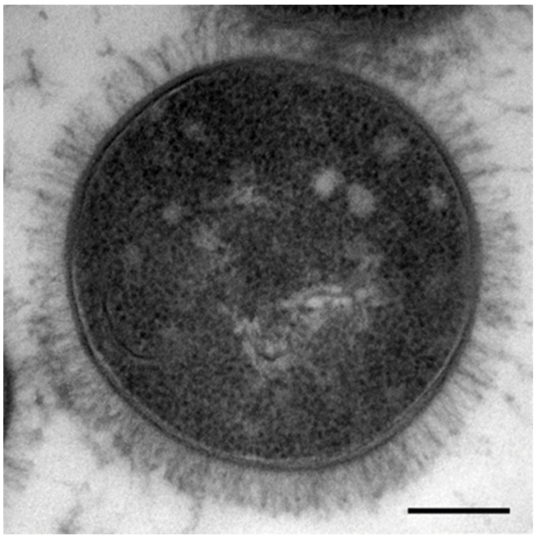

C 2016/00037/10/10

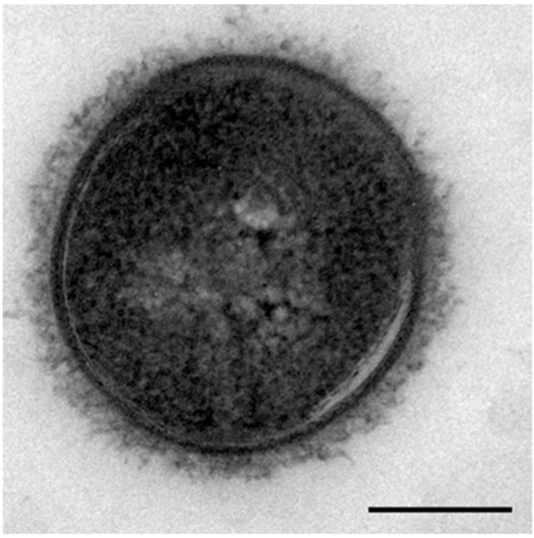

D 2015/03487/01/01

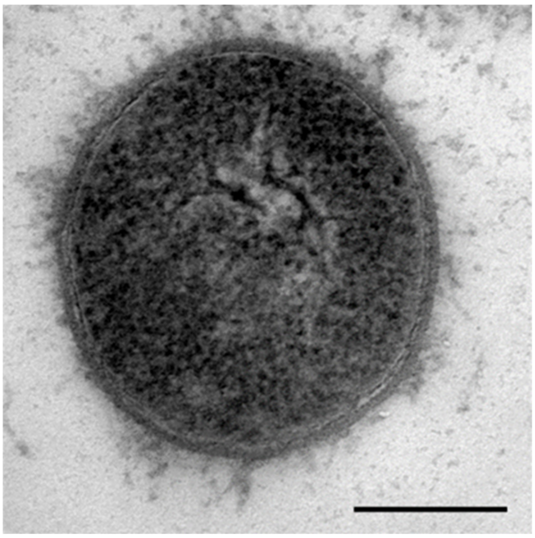

E 2016/03829/04/12

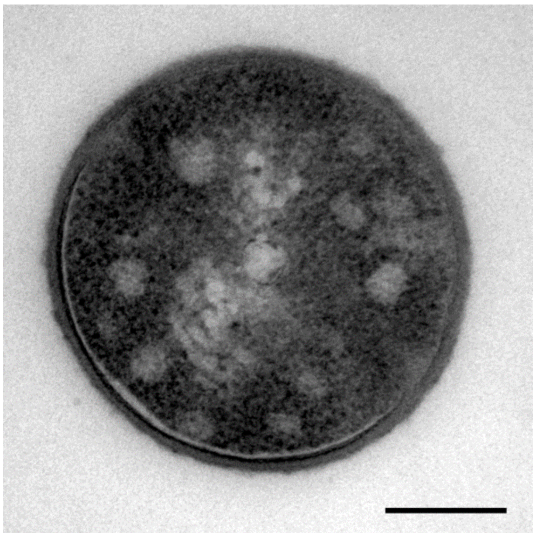

F 2016/03188/04/17

S3 Fig. TEM images of non-typable *S. suis* isolates representing an isolate with a well expressed capsule (A), a moderately well expressed capsule (B and C), a defective capsule (D), a very defective capsule (E) and no capsule (F). For details s. supporting information S6 Table. The scale corresponds to 0.2 µm.
