# Supplementary material for: Molecular typing of Streptococcus suis strains isolated from diseased and healthy pigs between 1996-2016
Source: PLoS One. 2019 Jan 17;14(1):e0210801. doi: 10.1371/journal.pone.0210801 (PMC6336254; doi:10.1371/journal.pone.0210801)
Supplement: S1 Table — (PDF) [file pone.0210801.s004.pdf]

S1 Table. Primers for *rec*N amplification integrated into two-step multiplex PCR by Okura et al. (J Clin Microbiol. 2014;52(5):1714-9.)

name	Sequence (5‘->3‘)	amplicon size bp
SsRECN_f	GCAATAGCAATGACTTGTGG	1247
SsRECN_r	ACAGGGGATTGAAGTAGCTG	
