# Supplementary material for: Molecular typing of Streptococcus suis strains isolated from diseased and healthy pigs between 1996-2016
Source: PLoS One. 2019 Jan 17;14(1):e0210801. doi: 10.1371/journal.pone.0210801 (PMC6336254; doi:10.1371/journal.pone.0210801)
Supplement: S2 Table — a nt: genetically non-typable by PCR. (PDF) [file pone.0210801.s005.pdf]

S2 Table. Serotypes of German *S. suis* isolates based on *cps* typing in collection A (1996-2004) and collection B (2015-2016), respectively, grouped by disease status

cps type	Invasive isolates				Pulmonary isolates				Carrier isolates			
	A		B		A		B		A		B	
	n	[%]	n	[%]	n	[%]	n	[%]	n	[%]	n	[%]
1 and 14	8	11,4	24	6,8	1	1,4	1	0,9	1	2,2	0	0
2 and 1/2	31	44,3	83	23,5	11	14,9	22	18,8	8	17,8	3	5,8
3	2	2,9	15	4,3	11	14,9	11	9,4	1	2,2	0	0
4	2	2,9	33	9,4	13	17,6	16	13,7	4	8,9	5	9,6
5	2	2,9	7	2	6	8,1	4	3,4	4	8,9	3	5,8
6	0	0	0	0	0	0	0	0	0	0	0	0
7	5	7,1	54	15,3	11	14,9	9	7,7	1	2,2	1	1,9
8	0	0	10	2,8	2	2,7	12	10,3	2	4,4	6	11,5
9	18	25,7	79	22,4	3	4,0	7	6	0	0	2	3,9
10	0	0	3	0,9	0	0	1	0,9	0	0	0	0
11	0	0	3	0,9	1	1,4	0	0	1	2,2	2	3,9
12	0	0	0	0	1	1,4	0	0	2	4,4	0	0
13	0	0	1	0,3	1	1,4	0	0	0	0	1	1,9
15	0	0	3	0,9	0	0	1	0,9	5	11,1	3	5,8
16	0	0	3	0,9	0	0	4	3,4	1	2,2	2	3,9
17	0	0	0	0	0	0	1	0,9	0	0	0	0
18	0	0	5	1,4	0	0	5	4,3	0	0	0	0
19	0	0	3	0,9	0	0	3	2,6	0	0	1	1,9
20	0	0	0	0	0	0	0	0	0	0	0	0
21	0	0	1	0,3	2	2,7	3	2,6	2	4,4	2	3,9
22	0	0	0	0	0	0	0	0	0	0	0	0
23	0	0	3	0,9	1	1,4	1	0,9	0	0	0	0
24	0	0	2	0,6	0	0	1	0,9	1	2,2	0	0
25	0	0	0	0	0	0	0	0	0	0	0	0
26	0	0	0	0	0	0	0	0	0	0	0	0
27	0	0	0	0	0	0	0	0	0	0	0	0
28	0	0	5	1,4	0	0	1	0,9	0	0	4	7,7
29	0	0	0	0	0	0	2	1,7	1	2,2	8	15,4
30	0	0	1	0,3	1	1,4	0	0	0	0	1	1,9
31	0	0	7	2	3	4,0	4	3,4	2	4,4	2	3,9
32	0	0	0	0	0	0	0	0	0	0	0	0
33	0	0	0	0	0	0	0	0	0	0	0	0
34	0	0	0	0	0	0	0	0	0	0	0	0
nt[a]	2	2,9	8	2,3	6	8,1	8	6,8	9	20	6	11,5
∑	70	100	353	100	74	100	117	100	45	100	52	100

[a] nt: genetically non-typable by PCR
