# Supplementary material for: Molecular typing of Streptococcus suis strains isolated from diseased and healthy pigs between 1996-2016
Source: PLoS One. 2019 Jan 17;14(1):e0210801. doi: 10.1371/journal.pone.0210801 (PMC6336254; doi:10.1371/journal.pone.0210801)
Supplement: S3 Table — a nt: genetically non-typable by PCR. b CNS: central nervous system. c Other: internal organs other than lungs. * 0.01<p≤0.05 significant difference between collection A and B or † within collection B between CNS and joint isolates. †† 0.001<p≤0.01 very significant difference within collection B between CNS and joint isolates *** p≤0.001 highly significant difference between collection A and B or ††† within collection B between CNS and joint isolates. (PDF) [file pone.0210801.s006.pdf]

S3 Table. Serotypes of German invasive *S. suis* isolates based on *cps* typing in collection A (1996-2004) and collection B (2015-2016), respectively, grouped by origin of isolation

cps type	Collection A			Collection B		
	CNS[b]	joint	other[c]	CNS[b]	joint	other[c]
1 and 14	4*	3	1	4*†††	17†††	3
2 and 1/2	25***	5	1	56***	17	10
3	2	0	0	14†	0†	1
4	1	0	1	27††	1††	5
5	1	0	1	6	1	0
6	0	0	0	0	0	0
7	3	1	1	36	14	4
8	0	0	0	8	2	0
9	12	1	5	57	13	9
10	0	0	0	3	0	0
11	0	0	0	1	1	2
12	0	0	0	0	0	0
13	0	0	0	1	0	0
15	0	0	0	3	0	0
16	0	0	0	1	1	1
17	0	0	0	0	0	0
18	0	0	0	4	1	0
19	0	0	0	3	0	0
20	0	0	0	0	0	0
21	0	0	0	0	1	0
22	0	0	0	0	0	0
23	0	0	0	3	0	0
24	0	0	0	1	0	1
25	0	0	0	0	0	0
26	0	0	0	0	0	0
27	0	0	0	0	0	0
28	0	0	0	4	0	1
29	0	0	0	0	0	0
30	0	0	0	1	0	0
31	0	0	0	5	0	2
32	0	0	0	0	0	0
33	0	0	0	0	0	0
34	0	0	0	0	0	0
nt[a]	0	2	0	3	2	3
∑	48	12	10	241	71	41

[a] nt: genetically non-typable by PCR
[b] CNS: central nervous system
[c] Other: internal organs other than lungs

* $0.01 < p \leq 0.05$ significant difference between collection A and B or † within collection B between CNS and joint isolates

†† $0.001 < p \leq 0.01$ very significant difference within collection B between CNS and joint isolates

*** $p \leq 0.001$ highly significant difference between collection A and B or ††† within collection B between CNS and joint isolates
