# Supplementary material for: Molecular typing of Streptococcus suis strains isolated from diseased and healthy pigs between 1996-2016
Source: PLoS One. 2019 Jan 17;14(1):e0210801. doi: 10.1371/journal.pone.0210801 (PMC6336254; doi:10.1371/journal.pone.0210801)
Supplement: S4 Table — a origin 1 = invasive; 2 = pulmonary; 3 = carrier. b courtesy of Dr. Hilde Smith, Central Veterinary Institute of Wageningen University Research; with sera for serotype 1/2 and 1–28. c no agglutination with serum 10 in repeated tests. d sequencing of the PCR-fragment showed 100%-identity with the wzy gene of another serotype 10 isolate in GenBank (https://www.ncbi.nlm.nih.gov/gene). (PDF) [file pone.0210801.s007.pdf]

S4 Table. Comparison of molecular and phenotypic serotyping for a representative selection of *S. suis* isolates

Isolate number	Origin[a]	wzy serotype	Serotype by co-agglutination[b]
2015/03720/01/04	1	1 or 14	14
2016/04142/03/03	1	1 or 14	1
2015/02554/03/05	1	2 or 1/2	2
2015/02137/08/05	1	3	3
2015/06009/01/01	1	4	4
2015/03598/02/07	1	5	5
2015/04296/01/01	1	7	7
2015/03050/01/02	1	8	8
2015/04208/02/03	1	9	9
2016/04630/01/01	1	9	9
2015/04246/01/09	1	10[d]	9[c]
2016/01289/03/04	2	10[d]	10, 9, 21, 22
2016/03610/03/03	1	11	11
2016/00402/06/06	2	12	12
2016/03962/03/06	2	13	13
2016/01741/09/30	2	15	auto-agglutination
2016/04444/01/02	1	15	15
2016/01728/01/01	1	16	16
2016/04146/12/12	2	17	17
2016/01717/01/01	2	18	18
2016/00843/01/01	1	19	19
2015/04639/01/02	2	21	21
2015/06010/02/02	1	23	23
2015/05085/01/01	1	24	24
2015/03881/02/02	2	28	auto-agglutination
2016/04031/01/02	3	28	28

[a] origin 1=invasive; 2=pulmonary; 3=carrier
[b] courtesy of Dr. Hilde Smith, Central Veterinary Institute of Wageningen University Research; with sera for serotype 1/2 and 1-28
[c] no agglutination with serum 10 in repeated tests
[d] sequencing of the PCR-fragment showed 100%-identity with the *wzy*-gene of another serotype 10 isolate in GenBank (https://www.ncbi.nlm.nih.gov/gene)
