# Supplementary material for: Molecular typing of Streptococcus suis strains isolated from diseased and healthy pigs between 1996-2016
Source: PLoS One. 2019 Jan 17;14(1):e0210801. doi: 10.1371/journal.pone.0210801 (PMC6336254; doi:10.1371/journal.pone.0210801)
Supplement: S5 Table — Number of isolates with/without genes for extracellular factor (epf), muramidase-released protein (mrp) and suilysin (sly) alone or in combination. (PDF) [file pone.0210801.s008.pdf]

S5 Table. Pathotype of selected serotypes (ST) based on *cps* typing of invasive, pulmonary and carrier *S. suis* isolates from collection A (1996-2004) and collection B (2015-2016). Number of isolates with/without genes for extracellular factor (*epf*), muramidase-released protein (*mrp*) and suilysin (*sly*) alone or in combination.

Collection A invasive isolates

	epf+ mrp+ sly+	*mrp+ sly+*	*mrp+*	*sly+*	*epf- mrp- sly-*	n
ST-1/ST-14	4	3	0	1	0	8
ST-2/ST-1/2	16	8	7	0	0	31
ST 3	0	2	0	0	0	2
ST4	0	2	0	0	0	2
ST 7	0	0	5	0	0	5
ST 8	0	0	0	0	0	0
ST9	0	17	0	0	1	18

Collection B invasive isolates

	epf+ mrp+ sly+	*mrp+ sly+*	*mrp+*	*sly+*	*epf- mrp- sly-*	n
ST-1/ST-14	17	7	0	0	0	24
ST-2/ST-1/2	42	14	22	5	0	83
ST 3	0	4	1	10	0	15
ST 4	0	31	0	2	0	33
ST 7	0	1	50	3	0	54
ST 8	0	2	0	8	0	10
ST9	0	64	0	11	4	79

Collection A pulmonary isolates

	epf+ mrp+ sly+	*mrp+ sly+*	*mrp+*	*sly+*	*epf- mrp- sly-*	n
ST-1/ST-14	0	1	0	0	0	1
ST-2/ST-1/2	0	2	8	1	0	11
ST 3	0	4	4	3	0	11
ST 4	0	12	0	1	0	13
ST 7	0	2	6	3	0	11
ST 8	0	1	0	1	0	2
ST 9	0	3	0	0	0	3

Collection B pulmonary isolates

	epf+ mrp+ sly+	*mrp+ sly+*	*mrp+*	*sly+*	*epf- mrp- sly-*	n
ST-1/ST-14	0	0	0	0	1	1
ST-2/ST-1/2	1	0	20	1	0	22

ST 3	0	2	0	9	0	11
ST 4	0	14	0	2	0	16
ST 7	0	0	7	1	1	9
ST 8	0	0	0	12	0	12
ST 9	0	5	0	1	1	7

Collection A carrier isolates

	epf+ mrp+ sly+	*mrp+ sly+*	*mrp+*	*sly+*	*epf- mrp- sly-*	n
ST-1/ST-14	0	1	0	0	0	1
ST-2/ST-1/2	2	1	5	0	0	8
ST 3	0	0	1	0	0	1
ST 4	0	3	0	0	1	4
ST 7	0	0	0	1	0	1
ST 8	0	0	0	2	0	2
ST 9	0	0	0	0	0	0

Collection B carrier isolates

	epf+ mrp+ sly+	*mrp+ sly+*	*mrp+*	*sly+*	*epf- mrp- sly-*	n
ST-1/ST-14	0	0	0	0	0	0
ST-2/ST-1/2	0	0	1	1	1	3
ST 3	0	0	0	0	0	0
ST 4	0	5	0	0	0	5
ST 7	0	0	0	0	1	1
ST 8	0	0	0	6	0	6
ST 9	0	0	0	0	2	2
