# Supplementary material for: Molecular typing of Streptococcus suis strains isolated from diseased and healthy pigs between 1996-2016
Source: PLoS One. 2019 Jan 17;14(1):e0210801. doi: 10.1371/journal.pone.0210801 (PMC6336254; doi:10.1371/journal.pone.0210801)
Supplement: S6 Table — a according to WILLENBORG et al. (2011). b origin 1 = invasive; 2 = pulmonary; 3 = carrier. c density:++++ extremely dense / +++ very dense / ++ moderately dense / + slightly dense / (+) very slightly dense /—no fuzzy material. d thickness:++++ ≥ 120 nm / +++ 90–119 nm / ++ 60–89 nm / + 30–59 nm / (+) ≤ 29 nm /—no fuzzy material. e score: average of density and thickness ++++ very well expressed capsule / +++ well expressed capsule / ++ moderately expressed capsule / + defective capsule / (+) very defective capsule /—no capsule. (PDF) [file pone.0210801.s009.pdf]

S6 Table. Characterization of the capsule for isolates non-typable by serological or molecular methods from collection B as revealed from TEM images.

Isolate number	Origin[b]	Capsule thickness [nm]	Interpretation		
			density[c]	thickness[d]	score[e]
St10[a]		100-150	++++	++++	++++
10Δ*ccpA*[a]		30-50	++	+	+
c10Δ*ccpA*[a]		60-90	+++	+++	+++
10Δ*cps*EF[a]		30-40	+	+	+
2016/04646/02/05	2	70-95	+++	+++	+++
2016/04144/09/09	2	80-130	++	++++	+++
2016/01183/05/05	1	70-100	++	+++	++
2016/03495/01/01	1	60-100	++	+++	++
2015/06015/09/25	1	50-70	++	++	++
2016/00037/10/10	2	70-85	++	++	++
2016/01991/01/01	1	70-80	++	++	++
2016/02985/02/03	1	50-65	++	++	++
2016/01635/01/01	2	60-75	+	++	+
2016/01739/04/09	3	25-40	++	+	+
2016/02027/01/02	3	20-35	++	+	+
2015/03487/01/01	1	30-50	+	+	+
2015/04406/01/02	2	25-40	+	+	+
2016/02253/01/01	1	30-55	+	+	+
2016/02284/04/04	2	25-35	+	+	+
2016/02992/01/02	1	50-70	+	+	+
2016/03290/20/20	3	30-45	+	+	+
2016/01990/01/01	3	25-55	(+)	+	(+)
2016/03829/04/12	3	40-55	(+)	+	(+)
2015/02796/01/09	2	20-25	+	(+)	(+)
2016/04145/13/13	2	20-25	+	(+)	(+)
2016/03188/04/17	3	0	-	-	-

[a] according to WILLENBORG et al. (2011)
[b] origin 1=invasive; 2= pulmonary; 3=carrier
[c] **density:**
++++ extremely dense / +++ very dense / ++ moderately dense / + slightly dense / (+) very slightly dense / - no fuzzy material
[d] **thickness:**
++++ ≥ 120 nm / +++ 90-119 nm / ++ 60-89 nm / + 30-59 nm / (+) ≤ 29 nm / - no fuzzy material
[e] **score:** average of density and thickness
++++ very well expressed capsule / +++ well expressed capsule / ++ moderately expressed capsule / + defective capsule / (+) very defective capsule / - no capsule
